# Accuracy of the Emergence Profile of Dental Implant Restorations using different gingival mask techniques

**DOI:** 10.4317/jced.64279

**Published:** 2026-07-29

**Authors:** Firas Abdulameer Farhan, Ali Jameel Abdul Sahib, Mustafa Mahdi Jassim, Bayan Saleem Khalaf, Abdalbseet Ahmad Fatalla

**Affiliations:** 1 PhD. Assistant Professor, Department of Prosthodontics, College of Dentistry, University of Baghdad, Bab Al-Muadham campus of the University of Baghdad, 1417, Baghdad, Iraq; 2 MSc. Assistant Professor, Department of Prosthodontics, College of Dentistry, University of Baghdad, Bab Al-Muadham campus of the University of Baghdad, 1417, Baghdad, Iraq; 3 Senior Lecturer, Department of Prosthodontics, College of Dentistry, University of Baghdad, Bab Al-Muadham campus of the University of Baghdad, 1417, Baghdad, Iraq; 4 MSc. Assistant Professor, Department of Prosthodontics, College of Dentistry, University of Baghdad, Bab Al-Muadham campus of the University of Baghdad, 1417, Baghdad, Iraq; 5 PhD. Professor, Prosthodontic Department, College of Dentistry, University of Baghdad, Bab Al-Muadham campus of the University of Baghdad, 1417, Baghdad, Iraq

## Abstract

**Background:**

Accurate reproduction of peri-implant soft tissue contours is essential for the esthetics, phonetics, hygiene, and long-term success of implant-supported restorations. Gingival mask (GM) techniques play an important role in reproducing the emergence profile (EP) and peri-implant soft tissue contour. This study evaluated and compared the accuracy of crown adaptation using conventional, 3D-printed, and digital GM workflows.

**Materials and Methods:**

Forty zirconia crowns were fabricated on implant standard abutments with a straight EP using a maxillary master model obtained from a patient missing left maxillary first molar. A single implant was placed using a guided surgical protocol and cone beam computed tomography. The crowns were divided into four groups (n=10) according to the GM technique: conventional, 3D-printed, adapted digital (DI-1), and non-adapted digital (DI-2). Crown adaptation and gap dimensions were evaluated using the digital subtraction method and a high-resolution desktop optical scanner. Scanning was performed for the abutment, internal crown surface, and the seated crown-abutment assembly. Measurements were obtained at buccal (B), palatal (P), mesial (M), and distal (D) points. Statistical analysis included Levene's test, one-way ANOVA, and Tukey's and Games-Howell post hoc tests (P 0.05).

**Results:**

The adapted digital GM group showed the lowest mean gap values at all measurement points, indicating superior adaptation accuracy, whereas the 3D-printed GM group showed the highest gap values. One-way ANOVA revealed significant differences among all GM groups at all measurement points (P=0.001). Post hoc analysis showed significant differences among most groups; however, no significant differences were found between the conventional and 3D-printed GM groups at point B or between the conventional and DI-2 groups at points P, M and D.

**Conclusions:**

GM techniques significantly influenced the accuracy of implant-supported restorations. The adapted digital GM workflow demonstrated superior adaptation and more accurate reproduction of peri-implant tissue contours and emergence profiles compared with the other techniques.

## Introduction

Dental implant-supported fixed restorations are widely accepted as a predictable and effective treatment modality for restoring partial and complete tooth loss. Successful implant restorations depend not only on osseointegration and mechanical stability, but also on the accurate reproduction of peri-implant soft tissue contours to achieve optimal esthetics, phonetics, hygiene access, and patient comfort (1). The gingival mask (GM) technique has therefore become an essential component of implant prosthodontics, as it reproduces the gingival contour created by restorations and enables the dental technician to transfer these contours to the definitive prosthesis accurately. In addition, the incorporation of cast verification procedures improves the passive fit of implant-supported prostheses while minimizing inaccuracies associated with impression copings and implant models (2 , 3). An ideal peri-implant emergence profile (EP) provides a smooth transition from the implant platform to the natural cervical contour of the restoration. It plays a critical role in soft tissue support, esthetic integration, phonetics, and periodontal health. Appropriate transmucosal contours facilitate positive tissue adaptation, prevent food impaction and air leakage during speech, and allow adequate access for oral hygiene maintenance. Conversely, excessive pressure on peri-implant tissues may compromise hygiene, prolong prosthesis insertion time, and negatively affect tissue health. GMs are therefore indispensable in both conventional and digital laboratory workflows because they simulate peri-implant tissues and guide the contouring of restoration margins and EPs. Traditionally, silicone-based GMs fabricated on stone casts from conventional impressions have been used for this purpose. However, one of the primary limitations of many conventional impression GM materials is the lack of long-term dimensional stability. Repeated insertion and removal during laboratory procedures can cause deformation, shrinkage, or tearing of the peri-implant tissue replica. These changes may reduce the accuracy of soft tissue simulation, ultimately affecting the fit and EP of the final restoration (4 - 6). Three-dimensional (3D-printed) GMs are considered to be one of the newest innovations in digital implant prosthodontics since they provide an accurate representation of peri-implant soft tissue contours and are seamlessly integrated into computer-aided design and computer-aided manufacturing (CAD/CAM) workflows. When compared with conventionally fabricated silicone GMs, additive manufacturing technologies propose several potential advantages, including high standardization of fabrication methods, reduced laboratory time, enhanced reproducibility and adaptation to fully digital restorative workflows. Furthermore, digital GMs facilitate communication between the clinician and dental laboratory technician by enabling the accurate and efficient transfer of peri-implant tissue contours throughout the design and fabrication of the final restoration (7). Although these technologies offer several advantages, significant limitations associated with currently available 3D-printed GM materials remain unresolved. Printable soft resins used as GM often exhibit inadequate elasticity, reduced tear resistance and poor dimensional stability resulting from repeated handling during laboratory procedures. Furthermore, the mechanical and viscoelastic properties of these materials fail to accurately replicate the resilience and biomechanical behavior of natural peri-implant tissues, potentially compromising the accuracy of emergence profile (EP) contouring and restoration adaptation. These limitations may ultimately affect the adaptation of implant-supported restorations to the peri-implant EP and gingival contours (5 , 8). Digital GM workflows have become an essential component of modern implant dentistry, particularly in the fabrication of implant-supported restorations. They integrate intraoral scanners (IOS) and CAD/CAM systems by combining digital data acquisition with computerized design and manufacturing processes. This approach enhances the accuracy, efficiency, and predictability of implant-supported prosthetic rehabilitation while improving patient comfort and communication between clinicians and dental laboratories. The IOS provides direct capture by scanning the oral cavity with a camera, while extraoral scanners provide indirect digitalization by scanning a cast resulting from conventional impression techniques. Accurate scanners, along with their associated software programs, enable precise fabrication of restorations without requiring impression materials or complex laboratory procedures (9 , 10). This in vitro study aimed to evaluate and compare the accuracy of crown adaptation to the peri-implant EP and gingival tissue contour using different GM fabrication techniques, including conventional impression methods, 3D-printed GMs and digital workflows.

## Materials and Methods

**Figure 1:**
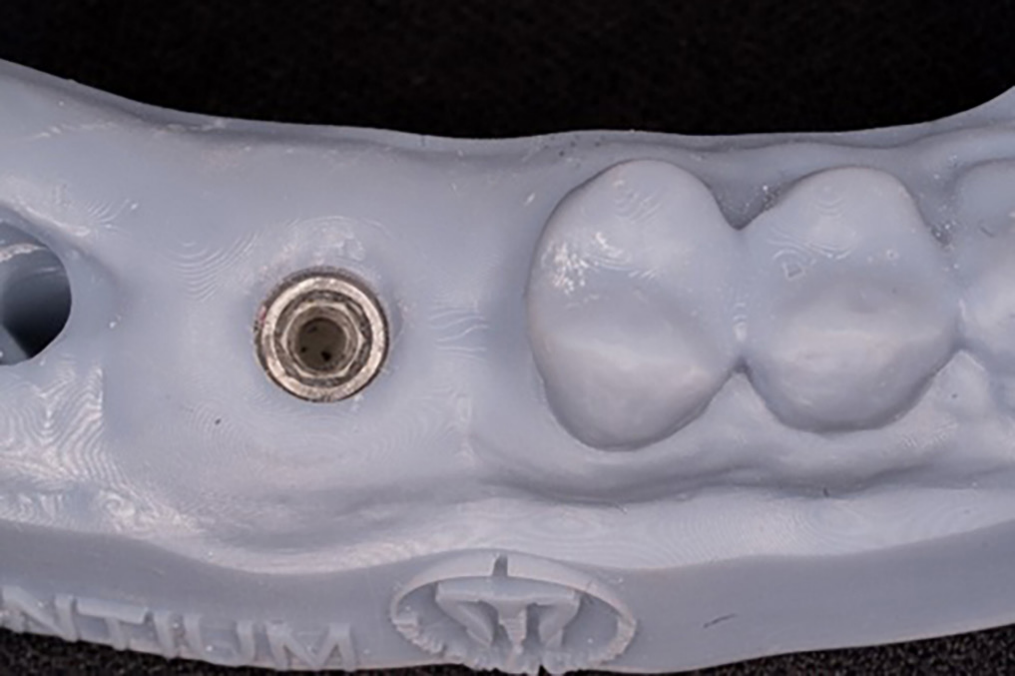
3D-printed master implant model with implant analogue.

- Preparation of the master implant model The study was conducted on a healthy 45-year-old female patient with a missing maxillary left first molar (tooth #26) and was approved ethically by the Research Ethics Committee of the University of Baghdad / College of Dentistry (Reference No. 1200; approved on 24 April 2026). A single dental implant was placed in the edentulous site using a surgical guided protocol and Dental Cone Beam Computed Tomography (CBCT) image. The osteotomy was prepared using the Dentium Implant Guide Kit (Dentium Co., Ltd., Korea) under the guidance of the manufacturer's protocol. Sequential drilling was performed, starting with a 2.4 mm pilot drill to a depth of 10 mm, followed by intermediate drills with diameters of 3.0 mm and 3.5 mm. The final osteotomy was completed using a 4.0 mm diameter drill. Subsequently, a screw-type root-form endosseous titanium implant (FXS4010 (D), Ti-6Al4V, Dentium Co., Ltd., Korea) was inserted into the osteotomy site and tightened using a manual ratchet to the manufacturer's recommended insertion torque to ensure primary stability. The prosthetic phase was initiated after a 3-month osseointegration period, followed by 10 days of peri-implant tissue healing using a gingival former to establish the EP. Subsequently, the implant site inside the patient was digitally captured using a high-resolution intraoral scanner (Medit i900, Medit Corp., South Korea). A printed replica was fabricated to serve as the master model using a photopolymer resin material (Formlabs Inc., USA) (Fig. 1).

1

**Figure 2:**
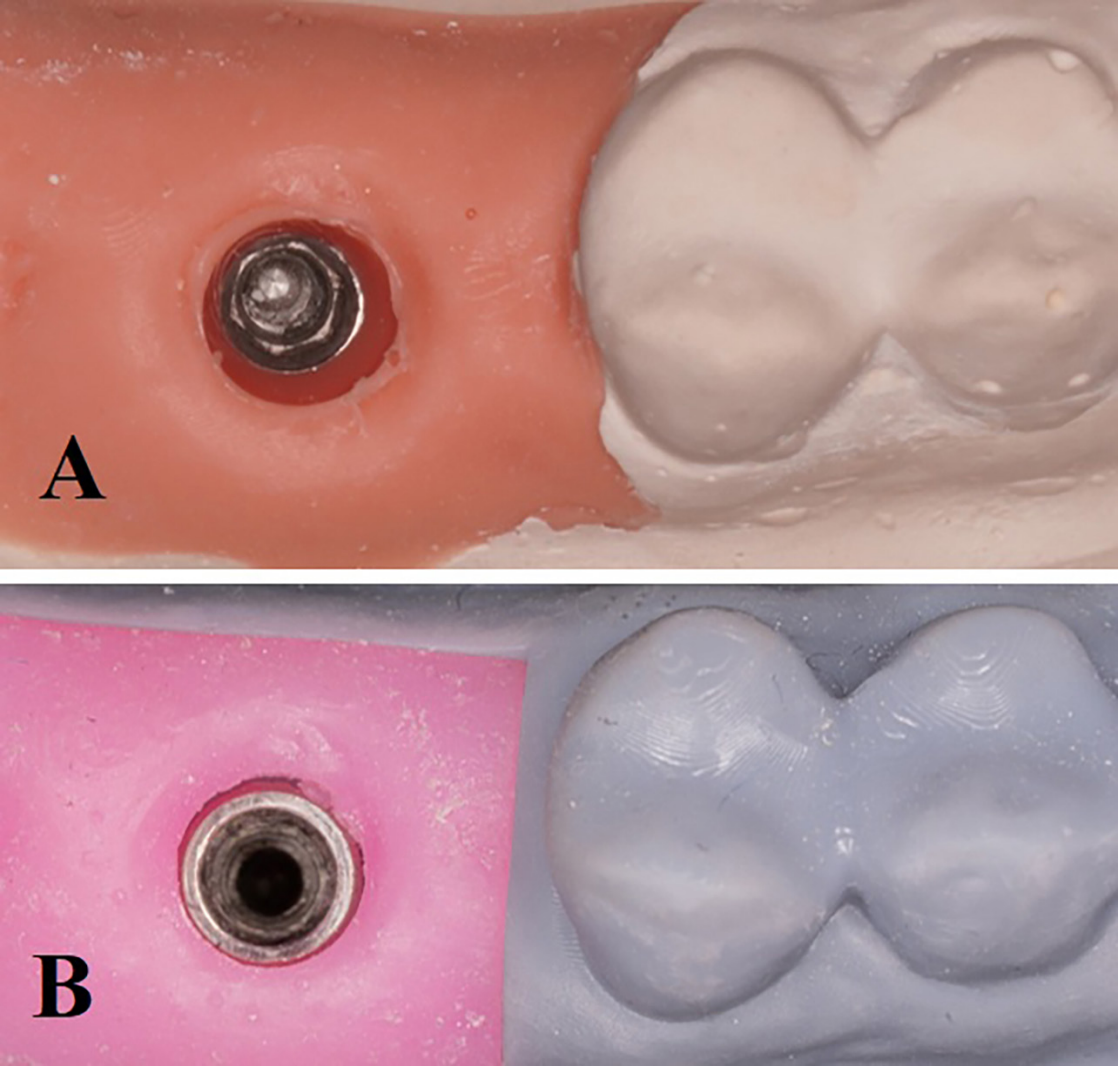
A) Conventional impression gingival mask and B) 3D Printed gingival mask.

- Samples preparation and grouping A total of 40 zirconia crowns were fabricated and designed over implant standard abutments with a straight EP on a maxillary master model for evaluation of marginal and internal fit, containing one implant analog (Dentium Co., Ltd., South Korea). The crowns were produced and classified into four groups (10 crowns per group) based on the type of GM fabrication techniques employed: Conventional Impression Gingival Mask Group A conventional impression was made using a custom tray and an elastomeric GM material (Gingifast Elastic; Zhermack, Italy) was used to reproduce the peri-implant tissue contour around the implant analog. The impression was poured with dental stone to obtain a working cast, which was subsequently scanned with a laboratory scanner. Zirconia crowns were then designed and fabricated using CAD/CAM workflow (Fig. 2A).

2

**Figure 3:**
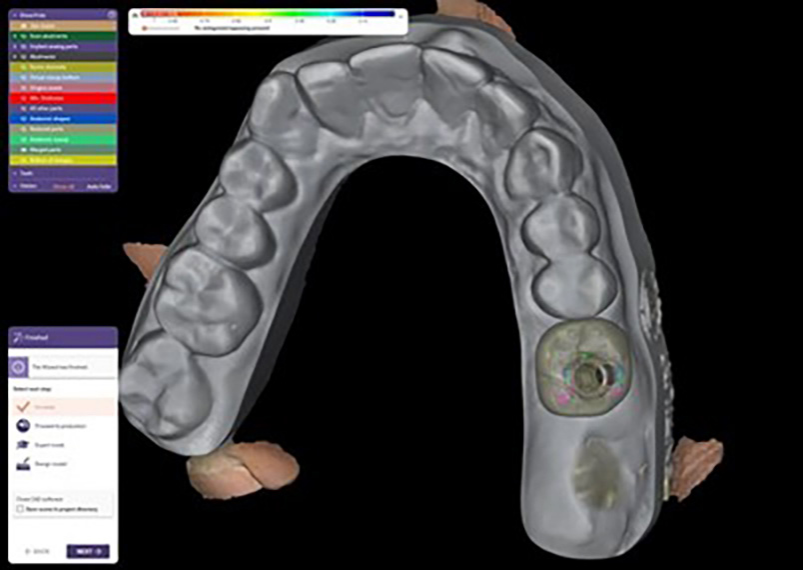
Digital gingival mask.

- 3D-Printed Gingival Mask Group The master model was digitally acquired using an intraoral scanner, and the digital dataset was used to design the working model and peri-implant tissue contours. The model was fabricated with a high-precision 3D printer (Form 3B; Formlabs Inc.), while the GM was printed using a flexible gingiva-colored resin. After assembly, the model was scanned with a laboratory scanner, and zirconia crowns were designed and milled using the same standardized CAD/CAM protocol as the other groups (Fig. 2B). - Digital Impression Group (DI) In this group, a virtual GM workflow was used to simulate peri-implant tissue outline. The master model was scanned using a high-resolution intraoral scanner (Medit i900; Medit Corp., Seoul, South Korea) to obtain the definitive digital impression. Within the CAD environment, virtual GMs were designed around the implant sites. The DI group was divided into two subgroups according to the GM design: Adapted group (DI-1): A 0.3-mm relief was created between the virtual GM and implant analog to optimize crown seating and adaptation. Non-adapted group (DI-2): The virtual GM was designed without relief or modification and served as the digital control. The finalized digital datasets were exported for CAD/CAM fabrication. Monolithic zirconia crowns were milled using the same materials and protocols as the conventional impression group to ensure standardization (Fig. 3).

3

**Figure 4:**
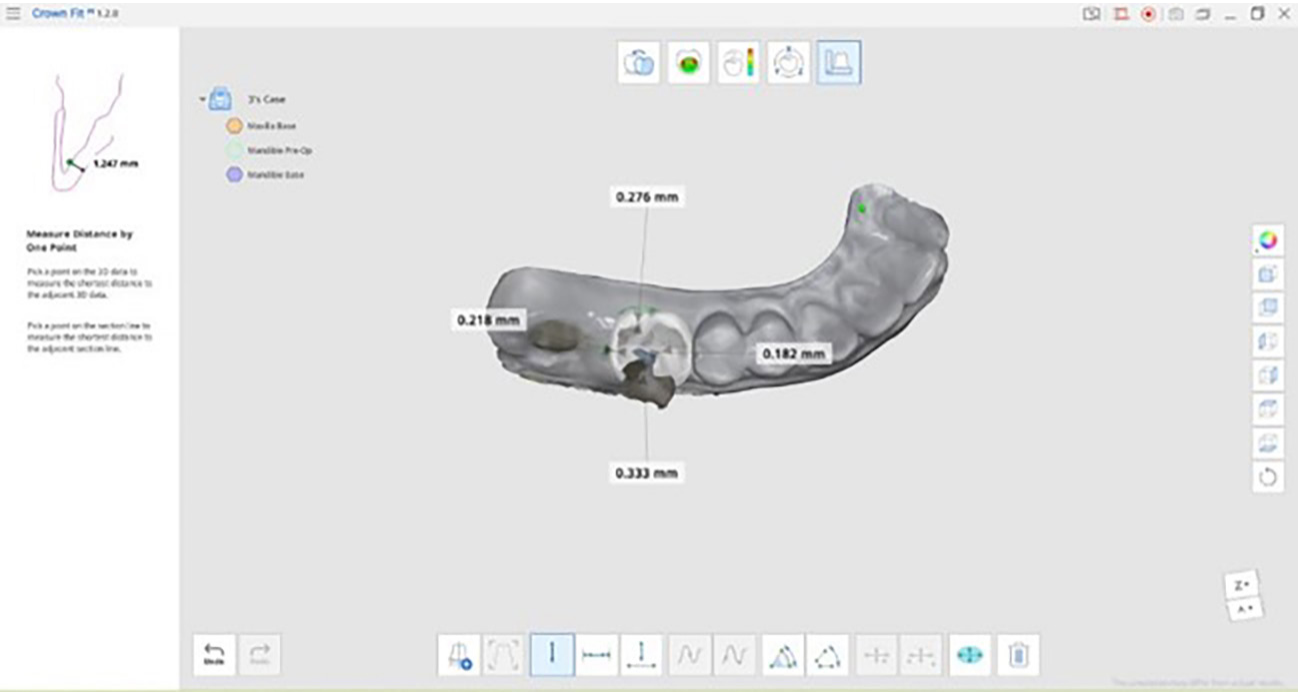
Digital subtraction method with four measurement points.

- Method of assessing restoration accuracy The digital subtraction method was used to assess the fitness and accuracy between a crown and the gingival margin in implant-supported restorations. Each crown and abutment assembly was scanned using a high-resolution intraoral scanner (Medit-i900 IOS, Medit Corp., Seoul, South Korea), with an accuracy of 10 µm and a scanning resolution of 10-15 µm. The scanning procedure included abutment, the internal surface of the crown and scanning the seated crown-abutment assembly. For standardization and subsequent digital processing, datasets from scans were converted into Standard Tessellation Language (STL) file format. The obtained files were loaded into three-dimensional evaluation software (Geomagic Studio 12, 3D System, USA) to evaluate the trueness of the crown and master model. For alignment using the "best-fit alignment" function in the software, which was used for the metrological assessment of the prosthodontic restorations. This software enabled accurate superimposition of the reference model and crown datasets using a best-fit alignment algorithm, permitting accurate evaluation of marginal and internal fit. - Digital Measurement Protocol Crown adaptation was evaluated in all groups using a standardized and quantitative method. Four measurement points were identified on each crown at key circumferential points around the implant abutment: buccal (B), palatal (P), mesial (M) and distal (D). These points were established on the digital model using anatomical reference axes to ensure consistent landmark positioning among all samples and experimental groups. At each designated point, the software measured the perpendicular linear distance between the internal surface of the crown margin and the finish line of the implant abutment on the master model. This distance was recorded as the marginal gap value for that specific site (Fig. 4).

4

Each measurement point was assessed three times to ensure measurement reliability and the mean value was calculated. All measurements were performed by a single calibrated examiner to minimize inter-operator variability. The mean marginal gap values recorded at the buccal, palatal, mesial and distal surfaces were subsequently used to compare the accuracy of crown adaptation among the four GM techniques. Statistical analysis Statistical Package for the Social Sciences (SPSS version 26, Chicago, Illinois, USA) was used for data description, analysis, and presentation. Levene's Test was used to assess the assumption of homogeneity of variances among the study groups. Parametric statistical tests were applied after confirming that the data followed a normal distribution (P > 0.05). One-way analysis of variance (ANOVA) was performed to evaluate differences among the groups, followed by Tukey's post hoc test for pairwise comparisons when equal variances were assumed and the Games-Howell Post Hoc Test when the assumption of homogeneity of variances was violated. Statistical significance was set at P 0.05.

## Results

Descriptive statistics included the mean gap values between the zirconia crown and the EP for the different GM techniques at each measurement point: buccal (B), palatal (P), mesial (M), and distal (D). The results showed that the adaptive digital GM group (DI-1) had the lowest mean gap values across all groups, indicating superior adaptation accuracy. In contrast, the 3D-printed GM group had the highest mean gap values as shown in Table 1.

Table 1

Levene's test demonstrated homogeneity of variances among the study groups. Parametric statistical tests were applied to the buccal (B) and palatal (P) measurement points after confirming normal data distribution (P > 0.05). One-way ANOVA was used to evaluate differences among the groups, followed by Tukey's post hoc test for pairwise comparisons when equal variances were assumed. For the mesial (M) and distal (D) measurement points, the Games-Howell post hoc test was applied when the assumption of homogeneity of variances was P 0.05. Statistical analysis using one-way ANOVA revealed significant differences among the GM groups at all measurement points (P=0.001), as presented in Table 2.

Table 2

These findings indicate that the type of GM had a significant effect on the gap values and overall fitness. The observed variations among the groups suggest that different GM fabrication techniques influence the accuracy and adaptation of the restoration. Multiple pairwise comparisons based on gap measurements demonstrated statistically significant differences among all GM groups. However, no significant differences were observed between the conventional and 3D-printed GM groups at point B and between the conventional and DI-2 GM groups at points P, M and D as shown in Table 3.

Table 3

The descriptive statistics and One-way ANOVA for overall dimensional accuracy at measurement points among fabrication techniques of GM showed a significant difference between groups at P<0.05 as shown in Table 4.

Table 4

## Discussion

Many studies in the literature have confirmed that implant-supported prostheses must meet several criteria to achieve long-term clinical success. Among these criteria, passive fit is considered one of the most critical factors influencing the longevity and stability of the restoration (11 , 12) Ensuring a passive and accurate prosthetic fit is crucial for peri-implant tissue health, functional stability, and long-term implant-supported restoration success. Achieving an accurate and passive fit is essential for minimizing both biological and mechanical complications associated with implant-supported prostheses. Biological complications, particularly peri-implant mucositis and peri-implant bone loss, may compromise peri-implant tissue health and ultimately affect implant survival. Furthermore, these biological complications may contribute to mechanical problems such as screw loosening, component wear, veneering material chipping, and fracture of implant or prosthetic components. Therefore, ensuring a passive and accurate prosthetic fit is fundamental for maintaining peri-implant tissue health, achieving functional stability, and enhancing the long-term success of implant-supported restorations (13 , 14). The fabrication of an accurately fitting implant-supported restoration begins with the production of a precise working cast that faithfully reproduces the intraoral implant position and surrounding peri-implant tissue contours. Any inaccuracies during the impression-making procedure may result in prosthetic misfit, unfavorable stress distribution, and potential prosthetic failure. Consequently, several implant impression techniques and materials have been developed to improve the accuracy of implant position transfer from the oral cavity to the laboratory, including conventional open-tray and closed-tray techniques as well as contemporary digital impression workflows (15 , 16). The selection of an appropriate implant impression technique represents the first critical step in minimizing dimensional discrepancies and improving prosthetic accuracy. An ideal impression technique should accurately transfer both implant position and peri-implant tissue outline while remaining clinically efficient, simple to perform, comfortable for the patient, and capable of reducing chairside and laboratory working time. Accurate impression procedures contribute directly to the fabrication of well-fitting restorations with improved esthetic, functional, and long-term clinical outcomes (16 - 18). The findings of the present study demonstrated significant differences among the investigated GM techniques regarding the accuracy of EP reproduction and restoration adaptation. The digital GM with adaptation (DI-1) exhibited the smallest marginal gap values at all measurement points (buccal, palatal, mesial, and distal) between the crown and the EP compared with the digital GM without adaptation (DI-2). Overall, the digital GM groups demonstrated superior fit and accuracy compared with the other groups, which may be attributed to their enhanced ability to reproduce peri-implant tissue contours and EPs more precisely during the fabrication of implant-supported restorations. The increased accuracy observed in the digital workflow may be explained by the ability of intraoral scanners to capture the exact implant position and surrounding gingival architecture with high precision. The acquired digital data are subsequently transferred to CAD/CAM systems for virtual restoration design, enabling more accurate replication of peri-implant soft tissue and improved adaptation of the definitive restoration to the EP (19). In addition, digital workflows support communication between the clinic and laboratory, reduce the need for conventional impression procedures, and minimize dimensional inaccuracies associated with traditional impression materials and stone casts. Digital GM workflows also facilitate the design of customized EPs, allowing better adaptation of implant restorations to peri-implant tissues and improving both esthetic and functional outcomes. Furthermore, the ability to digitally visualize and modify soft tissue contours enhances treatment planning and supports the fabrication of restorations with enhanced passive fit and tissue support. These workflows additionally facilitate clinical efficiency by reducing chairside and laboratory time, simplifying data storage and transfer, and enabling reproducible fabrication procedures (20 - 22). Despite these advantages, digital and CAD/CAM techniques still present several limitations. The accuracy of digital impressions depends largely on the performance of intraoral scanners and their ability to capture subgingival and critical peri-implant soft tissue details, particularly in deep or narrow EP areas. Errors occurring during intraoral scanning, digital design, or data processing may compromise the precise reproduction of peri-implant tissue contours and ultimately affect restoration adaptation (22 , 23). The 3D-printed GM group demonstrated higher gap dimension values than the other investigated groups, which may be related to the lower elasticity of printable GM materials compared with conventional silicone-based GMs. This reduced flexibility may compromise the accurate simulation of peri-implant tissue displacement during crown insertion and removal, especially in restorations with deep EPs. In addition, printable GM materials may not fully reproduce the biomechanical behavior of natural gingival tissues. Variations in material properties, including hardness, compressibility, and elastic recovery, may adversely affect restoration adaptation and limit accurate reproduction of the peri-implant EP (24 , 25). Several additional factors may influence the accuracy of 3D-printed GMs, including printer resolution, layer thickness, resin composition, printing orientation, and post-processing procedures such as washing and post-curing. Inaccuracies associated with any stage of the digital workflow may lead to dimensional distortion and compromise the accurate reproduction of peri-implant tissue contours. Moreover, photopolymer resins used for GM fabrication may undergo polymerization shrinkage during the curing process, potentially altering gingival architecture dimensions and adversely affecting restoration fit and adaptation. Consequently, the mechanical and physical properties of printed materials may differ from those of CI GM materials, limiting their ability to accurately simulate peri-implant soft tissue behavior (26 , 27). Furthermore, some 3D-printed GMs exhibit surface irregularities, including rough surface texture and visible layer lines resulting from the additive manufacturing process. These surface imperfections may negatively influence restoration adaptation, EP evaluation, and esthetic outcomes, often requiring additional finishing and polishing procedures to increase surface smoothness and clinical accuracy (28). Conventional impression GMs also present several limitations. The inherent rigidity of gypsum working casts may compromise the design of restorations intended to adapt to surrounding soft tissues. In addition, the physical properties of gypsum materials may result in dimensional distortion during fabrication, thereby reducing their ability to accurately reproduce the resilience and contour of natural gingiva. Conventional impression GM may also undergo distortion, tearing, or dimensional instability during fabrication and removal, potentially leading to inaccuracies in recording EP contours. Therefore, the incorporation of gingiva-mimicking materials into the working cast remains essential for improving soft tissue simulation and enhancing restorative outcomes (6 , 29). One of the major limitations of all GM techniques used in studies evaluating the accuracy of EPs in implant-supported restorations is the inability to completely reproduce the morphology and dynamic behavior of peri-implant soft tissues. None of the currently available GM materials can fully simulate the resilience, elasticity, and compressibility of natural gingival tissues under functional conditions.

## Conclusions

The accuracy and fitness of implant-supported restorations showed significant differences according to the type of GM fabrication technique used. The adapted digital GM group demonstrated superior adaptation and lower gap values compared with the other groups, indicating improved reproduction of the emergence profile and peri-implant soft tissue contours. However, no statistically significant difference was observed between the conventional and 3D-printed GM groups, suggesting that both techniques provided comparable levels of restoration adaptation.

## Figures and Tables

**Table 1:** Descriptive Statistical Analysis of Gap Dimension of Different Gingival Mask Techniques at Buccal (B), Palatal (P), Mesial (M), and Distal (D) Measurement Points in Millimeters (mm).

Measurement points	Gingival mask groups	N	Mean	Std. Deviation	

Point B	DI-1	10	0.34	0.064	
	DI-2	10	0.83	0.060	
	Conventional	10	1.17	0.121	
	3D Printed	10	1.24	0.156	
Point P	DI-1	10	0.29	0.041	
	DI-2	10	1.07	0.090	
	Conventional	10	1.10	0.135	
	3D Printed	10	1.68	0.24	
Point M	DI-1	10	0.45	0.054	
	DI-2	10	1.13	0.122	
	Conventional	10	1.16	0.120	
	3D Printed	10	2.01	0.192	
Point D	DI-1	10	0.32	0.059	
	DI-2	10	1.12	0.110	
	Conventional	10	1.184	0.118	
	3D Printed	10	1.775	0.431	

1

**Table 2:** One-Way ANOVA Analysis of Gap Dimension between Different Gingival Mask Techniques at Buccal (B), Palatal (P), Mesial (M), and Distal (D) Measurement Points.

Measurement points	Groups	Df	Mean Square	F	Sig. P<0.05
Point B	Between Groups	3	1.704	145.693	0.001
	Within Groups	36	0.012		
Point P	Between Groups	3	3.262	151.808	0.001
	Within Groups	36	0.021		
Point M	Between Groups	3	4.112	237.512	0.001
	Within Groups	36	0.017		
Point D	Between Groups	3	3.556	65.985	0.001
	Within Groups	36	0.054		

2

**Table 3:** Multiple Comparisons of Gap Dimension Using Tukey’s and Games-Howell Post Hoc Tests for Buccal (B), Palatal (P), Mesial (M), and Distal (D) Measurement Points among all Gingival Mask (GM) Groups.

Tukey’s Test					
Measurement points	(I) Groups	(J) Groups	Mean Difference (I-J)	Sig. P<0.05	

Point B	DI-1	DI-2	-0.493*	0.001	
		Conventional	-0.832*	0.001	
		3D Printed	-0.905*	0.001	
	DI-2	Conventional	-0.339*	0.001	
		3D Printed	-0.412*	0.001	
	Conventional	3D Printed	-0.073	0.439	
Point P	DI-1	DI-2	-0.779*	0.001	
		Conventional	-0.816*	0.001	
		3D Printed	-1.391*	0.001	
	DI-2	Conventional	-0.037	0.942	
		3D Printed	-0.612*	0.001	
	Conventional	3D Printed	-0.575*	0.001	
Games-Howell					
Point M	DI-1	DI-2	-0.685*	0.001	
		Conventional	-0.715*	0.001	
		3D Printed	-1.566*	0.001	
	DI-2	Conventional	-0.030	0.944	
		3D Printed	-0.881*	0.001	
	Conventional	3D Printed	-0.851*	0.001	
Point D	DI-1	DI-2	-0.797*	0.001	
		Conventional	-0.861*	0.001	
		3D Printed	-1.452*	0.001	
	DI-2	Conventional	-0.064	0.604	
		3D Printed	-0.655*	0.004	
	Conventional	3D Printed	-0.591*	0.008	

3

**Table 4:** Descriptive Statistics and One-Way ANOVA Analysis of Gap dimension among Different Gingival Mask Techniques.

Groups	N	Mean	Std. Deviation	F	Sig. P<0.05	

DI-1	10	1.67	0.891	78.651	0.001	
DI-2	10	4.22	0.311			
Conventional	10	4.87	0.898			
3D Printed	10	6.51	0.599			

4

## Data Availability

Declared none.
